# Supplementary material for: Prediction of Growth Characteristics and Migration Period of Spodoptera frugiperda (Lepidoptera: Noctuidae) According to Temperature
Source: Insects. 2022 Oct 2;13(10):897. doi: 10.3390/insects13100897 (PMC9604002; doi:10.3390/insects13100897)
Supplement: Supplementary file 1 [file insects-13-00897-s001.zip › insects-1905763-supplementary.pdf]

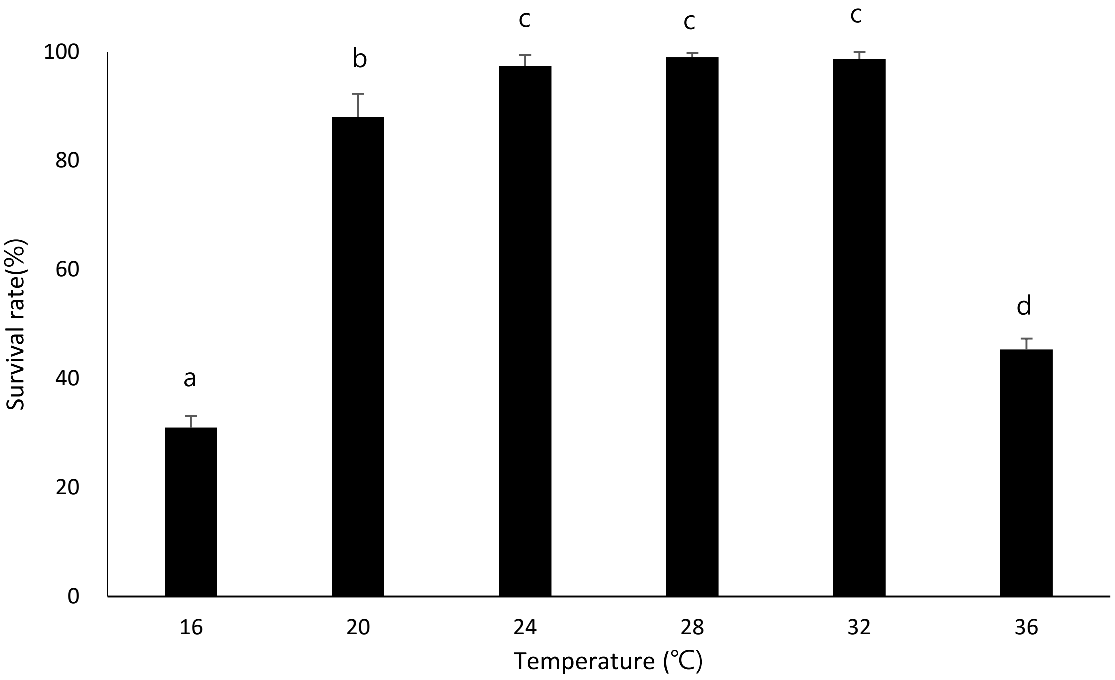

Figure **S1.** Survival rate of *Spodoptera frugiperda* larvae by temperature (16~36°C).

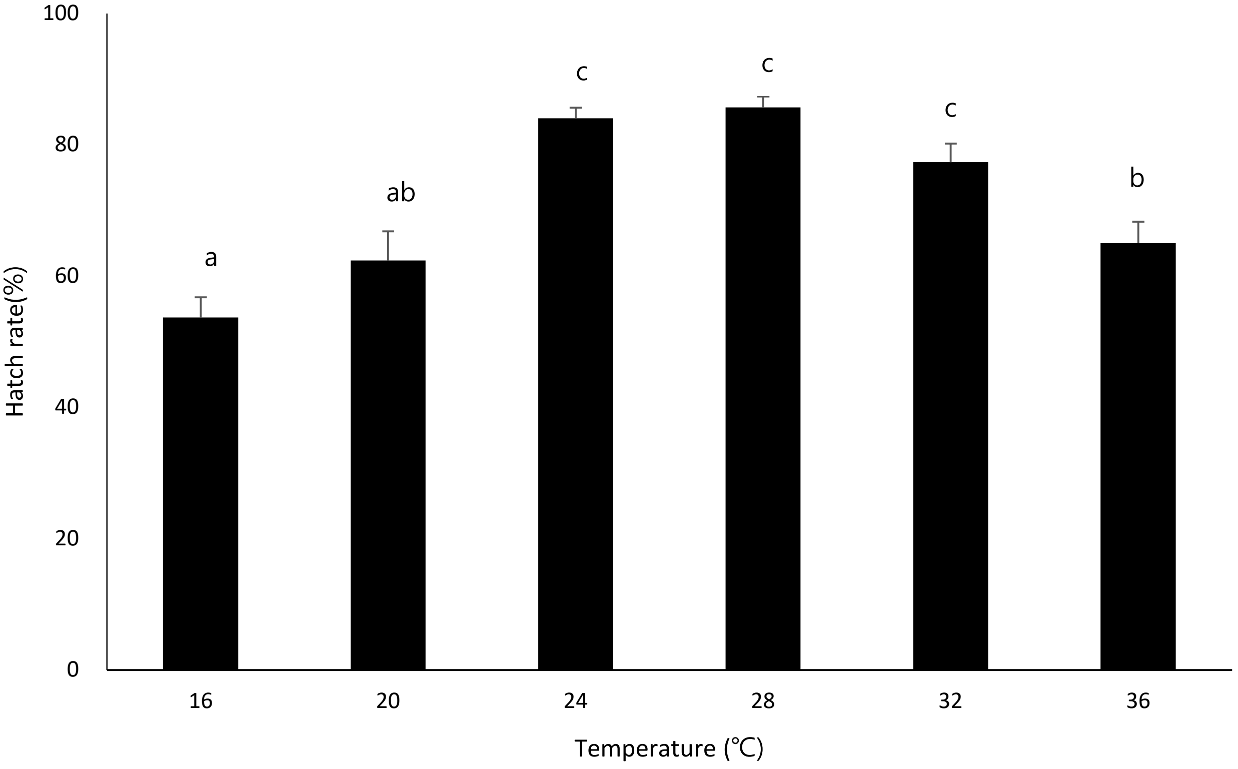

Figure S2. Hatch rate of *Spodoptera frugiperda* by temperature (16~36℃).
